# FAMILISM MODERATES RELATIONS BETWEEN SOCIAL NETWORK AND COGNITION IN MIDLIFE LATINE ADULTS

**DOI:** 10.1093/geroni/igae098.3230

**Published:** 2024-12-31

**Authors:** Adrienne Grudzien, Samantha Gonzales, Shanna Burke, Celine Cambara, Alexandra Briceno, Daniel Jimenez, Stephanie Scott, Sabrina Sales Martinez

**Affiliations:** Florida International University, Miami, Florida, United States; Florida International University, Miami, Florida, United States; Florida International University, Miami, Florida, United States; Florida International University, Miami, Florida, United States; Florida International University, Miami, Florida, United States; Florida International University, Miami, Florida, United States; Florida International University, Miami, Florida, United States; Florida International University, Miami, Florida, United States

## Abstract

Larger social network size, a measure of social isolation, has been linked to better cognitive function in older adults. Research indicates that stress-related immune and endocrine pathways may activate in socially isolated adults and negatively impact brain tissue. This study examined whether Latine adults reporting poorer social network quality but holding familial cultural values that prioritize closeness and loyalty in the family group experienced worse cognitive function. Cross-sectional multiple regression and moderation analyses were used to evaluate the influence of familism (via MACVS Obligations and Support scale) on relations between Lubben Social Network Scale score and National Alzheimer’s Coordinating Center cognitive domains in midlife Latine adults (n=51). Participants were predominantly female (92.16%), white (80.39%), and married or partnered (62.75%). The mean age of this sample was 52.84 (SD=4.89) years. Differential interaction effects were identified after adjusting for age, sex, race, income, and education level: familism strengthened relations between social network and episodic memory (β =.112, p = 0.019), and semantic memory (β =.059, p = 0.022) and weakened those with visuospatial (β =-.017, p = 0.044), executive function (β =-.04, p = 0.05), and verbal fluency (β = -.075, p = 0.046). These findings suggest that familism may be protective of visuospatial, executive function, and fluency domains, while semantic and episodic memory are more vulnerable in familism-aligned but socially isolated, aging Latine adults. Further evidence is necessary, however, to support the development of targeted interventions and support systems addressing cognitive health disparities in Latine adults.

